# Unipolar Barrier Photodetectors Based on Van Der Waals Heterostructure with Ultra‐High Light On/Off Ratio and Fast Speed

**DOI:** 10.1002/advs.202413844

**Published:** 2025-01-04

**Authors:** Suofu Wang, Xiuxiu Wang, Wenhui Wang, Tao Han, Feng Li, Lei Shan, Mingsheng Long

**Affiliations:** ^1^ Information Materials and Intelligent Sensing Laboratory of Anhui Province Key Laboratory of Structure and Functional Regulation of Hybrid Materials of Ministry of Education Institutes of Physical Science and Information Technology Anhui University 111 Jiu Long Road Hefei 230601 China; ^2^ School of Physics Southeast University Nanjing 211189 China; ^3^ Leibniz International Joint Research Center of Materials Sciences of Anhui Province Anhui University Hefei 230601 China

**Keywords:** heterostructure, long‐wave infrared, nBn, photodetector, ptse_2_

## Abstract

Unipolar barrier architecture is designed to enhance the photodetector's sensitivity by inducing highly asymmetrical barriers, a higher barrier for blocking majority carriers to depressing dark current, and a low minority carrier barrier without impeding the photocurrent flow through the channel. Depressed dark current without block photocurrent is highly desired for uncooled Long‐wave infrared (LWIR) photodetection, which can enhance the sensitivity of the photodetector. Here, an excellent unipolar barrier photodetector based on multi‐layer (ML) graphene (G) is developed, WSe_2_, and PtSe_2_ (G‐WSe_2_‐PtSe_2_) van der Waals (vdW) heterostructure, in which extremely low dark current of 1.61×10^−13^ A, a record high light on/off ≈10^9^ are demonstrated at 0 V. Notably, the device exhibits ultrafast response speed with rise time *τ*_r_ = 699 ns and decay time *τ*_d_ = 452 ns and high‐power conversion efficiency (*η*) of 4.87%. The heterostructure demonstrates a broadband photoresponse from 365 nm to LWIR 10.6 µm at room temperature. Notably, the G‐WSe_2_‐PtSe_2_ nBn device demonstrates high photoresponsivity (*R*) of 1.8 AW^−1^ with 10.6 µm laser at 1 V bias in ambient air. This unipolar barrier device architecture offers an alternative way for highly sensitive free space communication.

## Graphical abstract

A unipolar barrier photodetector uncooled long‐wave infrared nBn photodetector is demonstrated. The exciting experimental results include a record high light on/off ratio of ≈10^10^ and competitive fast speed with *τ*_r_ = 699 ns and *τ*_d_ = 452 ns are demonstrated with a 637 nm laser. This finds offers an alternative way for highly sensitive free space communication.

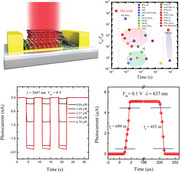

## 1 Introduction

Unipolar barriers allowing one carrier type to flow un‐impede and block the other carrier type, have been proposed to suppress dark current so that enable the infrared photodetectors to work at room temperature.^[^ ^1^ ^]^ Photodetectors using a unipolar barrier to block dark current without an unimpeded flow of photocurrent include two typical types of nBn (n‐type absorber, barrier layer and n‐type contact layer) and pBp (p‐type absorber, barrier layer and p‐type contact layer), in which the barrier layer is selected to block majority carriers electron and hole respectively.^[^ ^2^, ^3^, ^4^, ^5^, ^6^, ^7^, ^8^ ^]^ By carefully designing the band alignment, unipolar barrier heterostructure could be designed as a large band offset in either the conduction band or valence band to block majority carriers and zero band offset in the other band.^[^ ^3^ ^]^ Unipolar barriers have shown great potential ability in uncooled LWIR detection using HgCdTe^[^ ^3^, ^5^, ^6^, ^9^ ^]^ and InAsSb^[^ ^10^ ^]^ due to their wide tunable bandgap via varying alloy compositions. However, for those traditional bulk materials, many issues such as the complex epitaxial growth process, lattice mismatch inevitable interface defects, and the small minority carrier barrier impede the photocurrent and hinder the development of the high‐performance unipolar barrier photodetectors.^[^ ^11^ ^]^ Fortunately, using 2D layered materials built vdW heterostructure unipolar barrier detector can break the limit of lattice matching.^[^ ^12^, ^13^, ^14^ ^]^ Based on the 2D materials, broadband photoresponse with high performance from UV to the LWIR was demonstrated.^[^ ^15^, ^16^, ^17^ ^]^

In the application of narrow‐band gap infrared photodetectors, a low‐temperature environment is often required to reduce the dark current of the device, but this process is easily limited by Auger generation recombination (GR)^[^ ^2^, ^5^ ^]^ and Shockley‐Red Hall (SRH) processes,^[^ ^9^, ^18^, ^19^ ^]^ and it is difficult to operate manually at low temperature. This poses considerable difficulties for the application and detection of detectors in practical situations. To realize uncooled LWIR photodetection, the dark current should be well depressed. The unipolar barrier nBn heterostructures can effectively reduce the impact of Auger GR and SRH processes.^[^ ^18^, ^19^, ^20^ ^]^ Due to the dangling‐band‐free nature of 2D layered materials without limited lattice matching, vdW heterostructure with unipolar barrier band alignment can be designed.^[^ ^21^, ^22^, ^23^ ^]^ The surface leakage current is also further reduced.^[^ ^24^, ^25^ ^]^ In most photoconductive detectors, the dark current is too large to submerge the photocurrent or slow down the response time.^[^ ^26^ ^]^ And the photoconductive detector needs to work with the bias voltage.^[^ ^27^ ^]^ Therefore, self‐powered detectors without external forces will be the research trend in the future.^[^ ^28^, ^29^, ^30^ ^]^ PtSe_2_ is a narrow bandgap semiconductor with high mobility^[^ ^31^, ^32^, ^33^ ^]^ and excellent infrared detection ability,^[^ ^34^, ^35^, ^36^ ^]^ which could be used as infrared absorption material.

In this work, we designed a unipolar barrier vdW heterostructure G‐WSe_2_‐PtSe_2_ nBn photodetector. The device exhibited a record high light on/off ratio of ≈10^9^ and ultra‐fast photoresponse speed with *τ*_r_ = 699 ns and *τ*_d_ = 452 ns. A large conduction band offset *ΔE*_c_ and small valence band offset *ΔE*_v_ between WSe_2_ and PtSe_2_ enables the designing unipolar barrier nBn photodetector.

## 2 Results and Discussion

**Figure 1:**
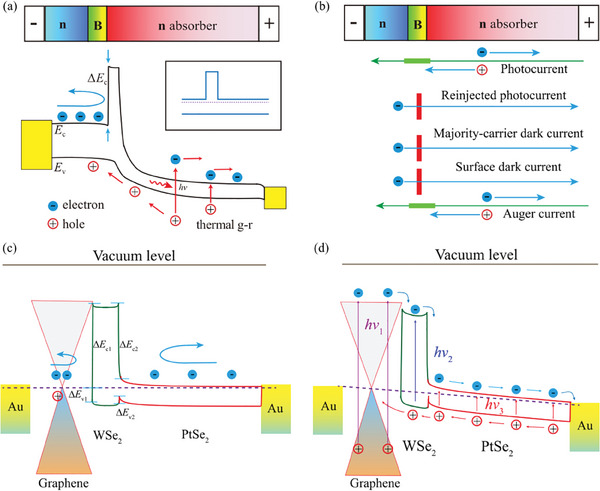
Energy band diagram and dark current of the unipolar barrier photodetectors. a) Schematic band diagram of nBn heterostructure with applied bias under the laser irradiation. Inset: a flat‐band condition in the electron barrier junction. b) Main contributors to the dark current in the nBn unipolar barrier photodetector. c) and d) Band diagram of nBn unipolar barrier photodetector G‐WSe_2_‐PtSe_2_ heterostructures without and with infrared illumination.

**Figure 2:**
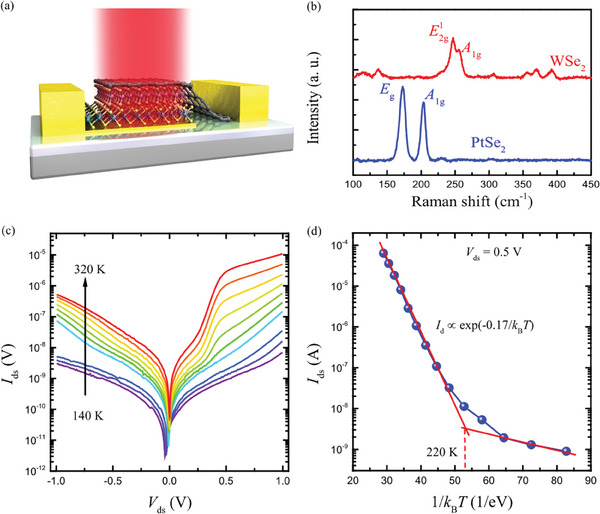
Photovoltaic characteristics of G‐WSe_2_‐PtSe_2_ heterostructures at room temperature. a) Schematic image of G‐WSe_2_‐PtSe_2_ vdW heterostructure photodetector. b) Raman spectra of PtSe_2_ and WSe_2_ nanoflakes. c) *I*–*V* curves of a typical G‐WSe_2_‐PtSe_2_ vdW heterostructure at different temperatures. d) Arrhenius plots the dark current unipolar barrier G‐WSe_2_‐PtSe_2_ vdW devices.

A unipolar barrier photodetector was designed as a large band‐offset barrier to block the majority carrier to depress the dark current. As shown in **Figure** **1**a, the band alignment of the nBn unipolar barrier photodetector is presented. The majority carrier was blocked by the high *ΔE*_c_ and the photocurrent can flow un‐impeded for the zero valence‐band offset. For the 2D vdW heterostructure, the bandgap of the n‐contact layer and the n‐absorber layer can be much different. The inset of Figure 1a shows the band structure of 2D vdW nBn heterostructure under the equilibrium condition. To illustrate the current blocking mechanisms, the positions of current generation and components of the current and flow through the nBn device are shown in Figure 1b. The photocurrent is major generated in the n‐absorber layer. The barrier layer blocks the reinjected photocurrent, majority carrier dark current, and surface dark current. The photocurrent and Auger current generated in the n‐absorber range. Using this band alignment, we design a G‐WSe_2_‐PtSe_2_ nBn vdW heterostructure. As shown in Figure 1c, the band profile of the G‐WSe_2_‐PtSe_2_ vdW heterostructure is presented. The large conduction band offset Δ*E*_c1_ and Δ*E*_c2_ plays a role as an electron barrier that impedes the majority carrier dark current. The photocarriers generated in the PtSe_2_ (h*v*_3_) could flow unimpeded as shown in Figure 1d. The device can realize broad‐band photoresponse from visible to the LWIR range. The schematic image of the G‐WSe_2_‐PtSe_2_ nBn heterostructure is shown in **Figure** **2**a. The infrared absorber PtSe_2_ flake is directly placed on a mirror electrode to shorten the photocarrier transport distance. The top electrode is in contact with the graphene. The Raman spectra of ML PtSe_2_ and WSe_2_ flakes are shown in Figure 2b. The up panel is the Raman spectrum of the ML WSe_2_. An obvious peak located at 250 cm^−1^ and with a shoulder peak at 260 cm^−1^ corresponds to the E_2g_^1^ and A_1g_ modes,^[^ ^37^, ^38^ ^]^ respectively. In the down panel of the PtSe_2_ Raman spectrum, two prominent vibrational modes of E_g_ and A_1g_ are observed posited at ≈175 and ≈205 cm^−1^, which is consistent well with the previous reports.^[^ ^31^, ^32^ ^]^ The Raman spectrum of the graphene layers is presented in Figure S1a (Supporting Information). Two peaks positioned at 1583 and 2715.2 cm^−1^ correspond to the G peak and the 2D peak,^[^ ^39^, ^40^ ^]^ respectively. The G peak corresponds to the E_2g_ phonon at the Brillouin zone center, and the 2D peak is the second order of the D peak. ^[^ ^39^ ^]^ The high intensity of the G peak and the unactivated D peak indicated the high quality of the graphene flake.^[^ ^41^ ^]^ The Raman spectra of the overlap region of the G‐WSe_2_‐PtSe_2_ heterostructure and the isolated graphene, WSe_2_, and PtSe_2_ are presented in Figure S1b (Supporting Information). The intensity of the isolated WSe_2_, and PtSe_2_ Raman peaks is much higher than that of the overlapped region, indicating that the high quality of the interface was realized. The atomic force microscope (AFM) was used to check the thicknesses of the G, WSe_2_, and PtSe_2_ flakes as shown in Figure S2 (Supporting Information). The thickness of G, WSe_2_, and PtSe_2_ flakes are 40, 15, and 25 nm respectively.

Next, we investigate the electric transport behavior of the G‐WSe_2_‐PtSe_2_ heterostructure device. We measured the temperature dependence Current‐Voltage (*I*–*V*). curves as shown in Figure 2c. As the temperature increased from 140 to 320 K, the current at 1 V bias increased from 7.0 nA to 10.6 µA. The dark current shows strong temperature dependence. The Arrhenius plot of the extracted temperature dependence current at 0.5 V bias is presented in Figure 2d. As the temperature increased, the current showed a very slow increase up to 220 K, indicating that the nBn heterostructure device can operate at a high temperature with a low dark current. A slope of ≈0.17 eV is obtained which is very close to the bandgap of the Mid‐wave infrared (MWIR) absorbing layer PtSe_2_ (0.1–0.3 eV).^[^ ^34^, ^42^ ^]^ The activation energy of the diffusion current is the bandgap *E*_g_, indicating that the thermal active diffusion current, which decreases with the temperature as *T* ^3^exp(‐*E*_g_/*k*_B_ *T*), limited dark current,^[^ ^2^, ^25^ ^]^ where T For the GR current, which is dominated by the generation of electrons and holes by the SRH traps in the depletion region, varies as *T* ^3^exp(‐*E*_g_/2*k*_B_ *T*).^[^ ^2^ ^]^ At high temperatures, the diffusion current is the major source of the dark current for both the nBn heterostructure device and the p‐n junction device. The unipolar barrier device exhibits a lower dark current than that of the p‐n junction device due to the absence of a depletion region without the SRH current. At the same dark current level, the nBn device can operate at a higher temperature than the p‐n junction device.

**Figure 3:**
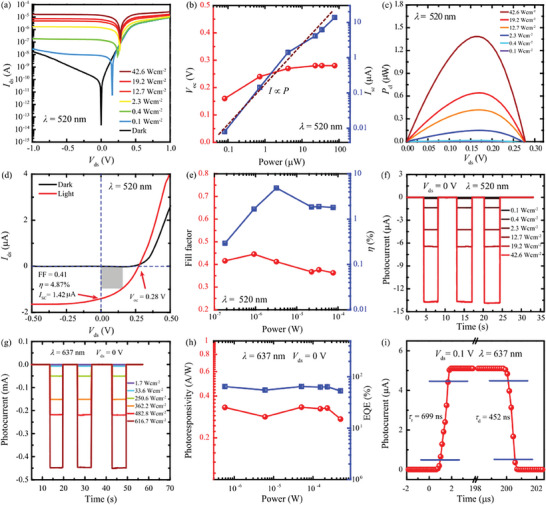
Optoelectronic characteristics of the G‐WSe_2_‐PtSe_2_ vdW unipolar barrier photodetectors in the visible spectrum range. a) Output characteristic curves of the G‐WSe_2_‐PtSe_2_ vdW unipolar barrier device under various powers of 520 nm laser illumination. b) The extracted *V*_OC_ and *I*_SC_ as a function of illumination power. c) Electrical power *P*_el_ versus bias voltage extracted from the device under different illumination powers of 520 nm laser. d) *I*–*V* curves of the G‐WSe_2_‐PtSe_2_ vdW unipolar barrier device with and without the illumination of 520 nm laser. e) Extracted fill factor and power conversion efficiency *η* as a function of illumination power of 520 nm laser. f) and g) Temporal photovoltaic response of the G‐WSe_2_‐PtSe_2_ vdW unipolar barrier device under different illumination powers of 520 and 637 nm lasers respectively. h) Incident light power dependence *R* and EQE as a function of illumination power at a bias of 0 V under 637 nm laser. i) The rise time of *τ*_r_ = 699 ns and decay time of *τ*_d_ = 452 ns of a G‐WSe_2_‐PtSe_2_ unipolar barrier vdW heterostructure photodetectors at 0.1 V bias.

Then, we investigate the photoresponse of the G‐WSe_2_‐PtSe_2_ heterostructure device in the visible range. We measured the output curves without and with various illumination power densities of the 520 nm laser, as shown in **Figure** **3**a. The obvious photovoltaic response was observed in this G‐WSe_2_‐PtSe_2_ heterostructure device. The extracted open circuit voltage (*V*_OC_) and short circuit current (*I*_SC_) as a function of incident light power are shown in Figure 3b. The *V*_OC_ shows a bit of incident light power dependence in the low light power region. As the light power increased from 82 nW to 76.7 µW, the *V*_OC_ increased from 0.16 to 0.28 V. The *I*_SC_ increased linearly as the incident light power increased and without showing any indication of saturation over the full measured range. The *I*_SC_ of 13.7 µA was realized as the incident light power density was 42.6 Wcm^−2^. We calculated the output electrical power (*P*_el_) with various incident light power, as shown in Figure 3c. As the incident light power increased, the *P*_el_ increased obviously. The highest *P*_el_ of 1.4 µW was obtained, indicating that this device with excellent photovoltaic response. To evaluate the performance of the photovoltaic response of the G‐WSe_2_‐PtSe_2_ heterostructure device, we calculated *η* and fill factor (FF). The power conversion efficiency is defined as *η* = *P*_el‐max_/*P*_I_, and *FF* = *P*_el‐max_/(*I*_SC_ *V*_OC_). Figure 3d shows the *I*–*V* curves of the G‐WSe_2_‐PtSe_2_ heterostructure device in the dark and under illumination. The highest *η* of 4.87% was obtained as the incident light power was 3.2 µW. The incident light power dependence *FF* and *η* are plotted in Figure 3e. In the low power range, the *η* increased from 0.29% to 4.87% as the incident light power increased. At a higher light power range, the *η* decreased to 1.85% and became stable. The *FF* showed a bit of fluctuation from 0.36 to 0.44. The energy conversion efficiency of this device is competitively compared with the other 2D materials heterostructures. Then we measured the time‐resolved photovoltaic response with various incident light power densities of 520 nm laser, as shown in Figure 3f. The extracted *R* and external quantum efficiency (EQE) as a function of light power is shown in Figure S3 (Supporting Information). The *R* is defined as the ratio of photocurrent (*I*_P_) to the effective illumination power (*P*_I_) incident on the device, and the EQE = *hcR*/(*e λ*), where *c*, *h*, *λ*, and *e*, are the speed of light, Planck's constant, the wavelength of the light and elementary electronic charge, respectively. The photovoltaic *R* of 0.32 AW^−1^ and corresponding EQE of 76.4% were demonstrated.

Next, we systematically investigated the photoresponse of the G‐WSe_2_‐PtSe_2_ heterostructure device with a 637 nm laser. The *I*–*V* curves with different incident light power densities were plotted in Figure S4a (Supporting Information). The extracted *V*_OC_ and *I*_SC_ as a function of incident light power are plotted in Figure S4b (Supporting Information). The *V*_OC_ exhibits a slight fluctuation from 0.24 to 0.27 V, and dependence on the light power weakly. The linear dependence of *I*_SC_ on the light power was observed. The slope in the low power range is smaller than that of a relatively higher light power. The fast increase in the *I*_SC_ in the higher power range could be attributed to the photothermal effect. The output electrical power of the device with different incident light power densities of 637 nm laser is plotted in Figure S4c (Supporting Information). The highest output electrical power of 29.3 µW was realized. The *I*–*V* curves of the G‐WSe_2_‐PtSe_2_ heterostructure device with and without illumination are shown in Figure S4d (Supporting Information). The *FF* of 0.24 and *η* of 2.6% were realized as the incident light power density was 616.7 Wcm^−2^. To check the photovoltaic response of the G‐WSe_2_‐PtSe_2_ heterostructure device. We measured the temporal photoresponse of the device with a 637 nm laser at 0 V bias as shown in Figure 3g. The photocurrent of 0.45 mA was obtained at the illumination power density of 616.7 Wcm^−2^. The logarithmic coordinate of the absolute value of the photocurrent with a 637 nm laser is shown in Figure S5a (Supporting Information). At 0 V, the dark current is 1.6 × 10^−13^ A. As the incident light power density was 616.7 Wcm^−2^, the current is up to 4.7 × 10^−4^ A. An ultrahigh light on/off ratio *I*_on_/*I*_off_ of ≈10^9^ was realized at 0 V bias. The extracted *R* and EQE versus light powers are presented in Figure 3h. The *R* and EQE exhibit weak light power dependence. In the full measured light power range, the *R* exhibits a bit fluctuation from 0.27 to 0.33 AW^−1^, corresponding EQE ranges from 53.1% to 64.7%. The photoresponse speed is one of the most important parameters for a photodetector. We measured the photoresponse speed of the G‐WSe_2_‐PtSe_2_ nBn heterostructure device at 0.1 V bias, as shown in Figure 3i. The rise time, from 10% to 90% of stable photocurrent, of 699 ns, and decay time, from 90% to 10% of stable photocurrent of 452 ns were demonstrated. The photoresponse speed of this G‐WSe_2_‐PtSe_2_ nBn heterostructure device is one of the most competitive compared with 2D material‐base photodetectors. The fast photoresponse speed could be attributed to the vertical device configuration with atomic short photocarrier transport distance and the bottom mirror electrode's high photocarrier collection efficiency. The temporal photoresponse of the G‐WSe_2_‐PtSe_2_ nBn device at 1V bias is presented in Figure S5b (Supporting Information). The good repeatability and sharply increased/decreased as the light turned on/off are observed. A high photocurrent of 461.2 µA was realized with 50.0 Wcm^−2^ illumination power density at 1 V bias. Extracted *R* and EQE as a function of incident light power at 1 V bias is presented in Figure S5c (Supporting Information). The *R* exhibits weak light power dependence and a bit of fluctuation from 4.5 to 5.2 AW^−1^. Corresponding EQE is fluctuated from 883.3% to 1007.5%. In the short‐wave communication spectral range, the photocurrent of the G‐WSe_2_‐PtSe_2_ nBn device with various power densities of 1650 nm laser at 1 V bias is plotted in Figure S6a (Supporting Information). The photoresponse speed with a 1650 nm laser is presented in Figure S6b (Supporting Information). In the communication spectral range, the device exhibited fast speed with a rise time of 0.3 ms and decay time of 0.4 ms. The photoresponse of the G‐WSe_2_‐PtSe_2_ nBn device with 365 nm illumination at 1 V bias is shown in Figure S7 (Supporting Information). The fast response speed with 365 nm light was observed.

**Figure 4:**
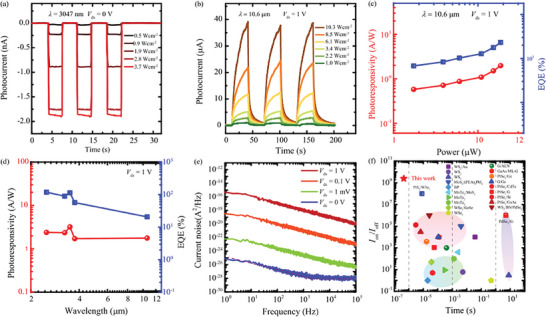
Photoresponse of G‐WSe_2_‐PtSe_2_ vdW unipolar barrier device in MWIR spectral range. a) Temporal photoresponse of the G‐WSe_2_‐PtSe_2_ vdW unipolar barrier device under various illumination powers of 3047 nm MWIR laser. b) Time‐resolved photoresponse of the G‐WSe_2_‐PtSe_2_ vdW unipolar barrier device with different power of LWIR 10.6 µm laser at 1 V bias. c) Calculated *R* and EQE versus light power 0f 10.6 µm laser at 1 V bias. d) Noise current power density of the measured device with different biases. e) Wavelength dependence *R* and EQE of the G‐WSe_2_‐PtSe_2_ vdW at 1 V bias. f) Comparison of light on/off ratio and response time among reported 2D material photodetectors at room temperature.

Then, we investigate the performance of the G‐WSe_2_‐PtSe_2_ nBn heterostructure device operation in the MWIR spectral range. In the MWIR spectral range, we measured the temporal photoresponse of the device with different power densities of 3047 nm laser at 0 V biases, as shown in **Figure** **4**a. The photoresponse speed at 0 V bias is very fast, and the photocurrent is a few nanoamperes. The low photocurrent could be attributed to the small valance band shift Δ*E*_v2_, as shown in Figure 1c, which impeded the photogenerated hole flow to the graphene. The temporal photoresponse of the G‐WSe_2_‐PtSe_2_ nBn device at 1 V bias with various light power densities of the MWIR 2500, 3250, 3500, and 3750 nm are present in Figure S8 (Supporting Information). With 6.2 Wcm^−2^ incident light power density of the 2500 nm laser, the photocurrent up to 29.2 µA was realized. The calculated *R* and EQE at 1 V biases of the MWIR spectral range are shown in Figure S9 (Supporting Information). In the MWIR spectral range, the increasing tendency of *R* as the light power increased was observed. The *R* of 3.2 AW^−1^ was realized with the 3500 nm laser.

Next, we investigate the performance of the G‐WSe_2_‐PtSe_2_ nBn device in the LWIR spectral range. The time‐resolved photoresponse of the G‐WSe_2_‐PtSe_2_ nBn device with different power densities of the 10.6 µm laser at 1V bias is presented in Figure 4b. As the light power was 10.3 Wcm^−2^, the photocurrent of 39.2 µA was demonstrated. Figure 4c shows the extracted *R* and EQE as a function of incident light power. At 1 V bias, the *R* of 1.8 AW^−1^, and corresponding EQE of 20.7% were realized, indicating that the G‐WSe_2_‐PtSe_2_ nBn device process has excellent uncooled LWIR detection ability. The wavelength dependence *R* and EQE from MWIR to LWIR at 1 V bias is shown in Figure 4d. From the MWIR and the LWIR spectral range, the *R* showed a bit of decreasing tendency as the wavelength increased. The extracted photovoltaic *R* and EQE versus wavelength of incident light from visible to the MWIR is shown in Figure S10a (Supporting Information). The photoresponsivities in the visible range are much higher than that in the MWIR range. This could be attributed to the interface barrier which impedes the low‐energy photocarriers considerably. For the higher energy photocarrier, they can overcome the barrier and flue to the electrode to become the photocurrent. To evaluate the ability of the device who pick up the signal from the background noise, we measured the current noise power spectrum of the device applied with various biases, as shown in Figure 4e. At the low bias, the current noise power density spectra *S*(*f*) were depressed obviously. At 0 V bias, the 1/*f* noise dominated the current noise contribution as the frequency increased to 2 kHz. As the frequency is larger than 2 kHz, the current noise power density spectrum becomes independent of the frequency (white noise). The noise equivalent power (NEP) and specific detectivity (D^*^) are the most important figure‐of‐merits for the detector which are used to evaluate the sensitivity of a detector. NEP and D* can be calculated using the following formulas NEP = *i*_n_/*R*, and D^*^ = *A* ^1/2^ *R*/*i*_n_, where *A* is the effective area of the measured device. The *i*_n_ can be obtained by calculate the mean‐square‐roots using the formula of <*i*_n_^2^ >^1/2^ = $\frac{1}{B}\int_{0}^{B}S\left(f\right)df$, where *B* is the measured electric bandwidth. At 0 V bias, the <*i*_n_^2^ >^1/2^ = 1.12 × 10^−14^ AHz^−1/2^ was obtained. The NEP and D^*^ of the G‐WSe_2_‐PtSe_2_ nBn heterostructure device versus wavelength are plotted in Figure S10b (Supporting Information). In the visible range, the NEPs lower than 0.7 pWHz^−1/2^, and the D^*^ of 6.4 × 10^10^ cmHz^1/2^W^−1^ were demonstrated. As the wavelength increased to the MWIR 2611 nm, the D* was higher than the commercial thermistor bolometer of 10^8^ cmHz^1/2^W^−1^. To compare the performance of this G‐WSe_2_‐PtSe_2_ nBn heterostructure device, we have summarized the light on/off ratio and photoresponse speed of 2D material‐based photodetectors as shown in Figure 4f. Our device with both a high light on/off ratio and fast speed. The performance of this nBn device and other 2D materials photovoltaic detectors are summarized in Table S1 (Supporting Information) for comparison. The dynamic dark current with different biases is presented in Figure S11. The dark current was stable without obvious fluctuation indicating that the high quality of the device was obtained.

**Figure 5:**
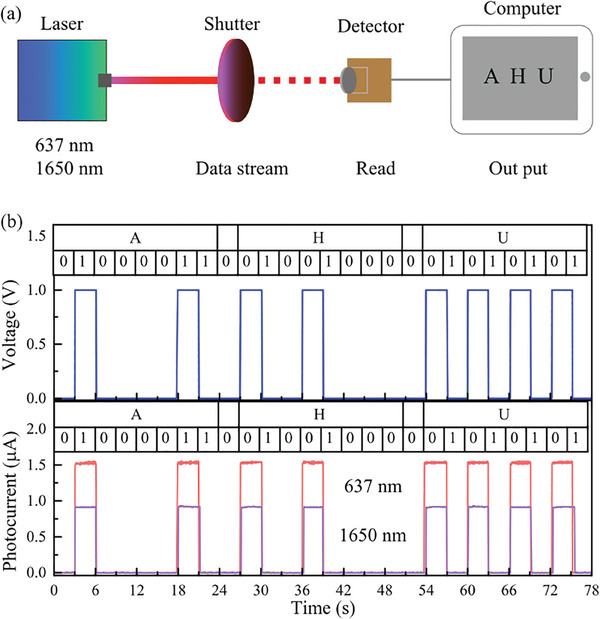
Optical communication system based on G‐WSe_2_‐PtSe_2_ nBn photodetector. a) Schematic drawing of the optical communication system of the G‐WSe_2_‐PtSe_2_ nBn photodetector. b) The input and output signals transmit the ASCII code “AHU” in the G‐WSe_2_‐PtSe_2_ optical communication system.

Finally, the G‐WSe_2_‐PtSe_2_ nBn device shows great potential application in optical communication for the fast photoresponse speed and ultra‐high light on/off ratio. As shown in **Figure** **5**a, a visible 637 nm laser and a communication band of the 1650 nm laser were used as light sources. The input message was converted into American Standard Code for Information Interchange (ASCII) codes by changing the switch on/off the light using a timing‐controller shutter. A G‐WSe_2_‐PtSe_2_ nBn heterostructure device was used as a signal receiver. The signal output from the detector was transmitted to a terminal computer through a source‐measure unit. Figure 5b shows the input ASCII signals of the letter “AHU” encoded by a shutter. The received signal at the detector is shown in Figure 5c. The good square waves matched well with the input information indicating that the high‐quality information transmission was realized.

## 3 Conclusion

We design a unipolar barrier photodetector based on a G‐WSe_2_‐PtSe_2_ nBn vdW heterostructure. A record high light on/off ratio of ≈10^9^, a high energy conversion efficiency of 4.87%, and an ultrafast photoresponse speed with *τ*_r_ = 699 ns and *τ*_d_ = 452 ns were demonstrated. The device demonstrated ultrabroad band detection from 365 nm to 10.6 µm. Notably, the device exhibits excellent uncooled LWIR detection ability with a high *R* of 1.8 AW^−1^ was realized. Additionally, based on the fast photoresponse speed and ultra‐high light on/off ratio, high signal‐to‐noise information transmission was demonstrated.

## 4 Experimental Section

### Device Fabrication

The bottom electrodes were patterned using the ultraviolet lithography on the silicon substrate covered by 300 nm SiO_2_. Then 5 nm Cr and 25 nm Au were deposited by thermal evaporation. Graphene, WSe_2_, and PtSe_2_ nano‐flakes were prepared by traditional mechanical exfoliation. The G‐WSe_2_‐PtSe_2_heterostructure was fabricated by the dry transfer technique. The top electrodes were prepared by electron beam lithography and followed by a thermal evaporation deposition of 10 nm Cr and 50 nm Au.

### Materials Characterization

The thicknesses of graphene, WSe_2_, and PtSe_2_ of the nanosheets were checked by atomic force microscopy (HITACHI, AFM 5500M). Raman spectra were measured using a 532 nm laser as an excitation source by a Renishaw Invia Reflex system.

### Electrical and Photoresponse Measurements

Electrical transport measurements were carried out using a dual‐channel digital source meter (Keithley 2636B). A homemade wavelength‐tunable multichannel MWIR laser (2.5–4.2 µm) laser was used to investigate the performance of the detector in the MWIR ranges. The diameter of the MWIR laser beam was ≈3 mm. The performance of the LWIR 10.6 µm was investigated using a CO_2_ laser source. From 405 to 1650 nm, the light sources used semiconductor fiber lasers (Thorlabs), which were focused on the device using a 20× objective lens. The *I*–*V* curves at different temperatures were measured by a physical property measurement system (PPMS, Quantum Design).

## Conflict of Interest

The authors declare no conflict of interest.

## Author Contributions

S.W. and X.W. contributed eqully to this work. M. L. conceived the project and designed the experiment. S. W. and X. W. fabricated the device and measurements. S. W., X. W., and W. W. carried out the material characterization, and M. L. and S. W. analyzed the data analysis and wrote the manuscript. All the authors reviewed and commented on the manuscript.

## Supporting information



Supporting Information

## Data Availability

The data that support the findings of this study are available from the corresponding author upon reasonable request.
