# Supplementary material for: Unipolar Barrier Photodetectors Based on Van Der Waals Heterostructure with Ultra‐High Light On/Off Ratio and Fast Speed
Source: Adv Sci (Weinh). 2025 Jan 4;12(8):2413844. doi: 10.1002/advs.202413844 (PMC11848579; doi:10.1002/advs.202413844)
Supplement: Supplementary file 1 — Supporting Information [file ADVS-12-2413844-s001.docx]

**Supporting Information**

**Unipolar Barrier Photodetectors Based on van der Waals Heterostructure with Ultra-high Light On/Off Ratio and Fast Speed**

Suofu Wang,^1+^ Xiuxiu Wang,^1+^ Wenhui Wang,^2^ Tao Han,^1*^ Feng Li,^1^ Lei Shan,^1, 3*^ and Mingsheng Long^1, 3*^

1. Information Materials and Intelligent Sensing Laboratory of Anhui Province, Key Laboratory of Structure and Functional Regulation of Hybrid Materials of Ministry of Education, Institutes of Physical Science and Information Technology, Anhui University, 111 Jiu Long Road, Hefei 230601, China

2. School of Physics, Southeast University, Nanjing 211189, China

3. Leibniz International Joint Research Center of Materials Sciences of Anhui Province, Anhui University, Hefei 230601, China.

Corresponding authors: M. L. ([longms@ahu.edu.cn](mailto:longms@ahu.edu.cn)), L. S. ([lshan@ahu.edu.cn](mailto:lshan@ahu.edu.cn)), and (than@ahu.edu.cn)

**Keywords:** Heterostructure, Long-wave infrared, Photodetector, nBn, PtSe_2_


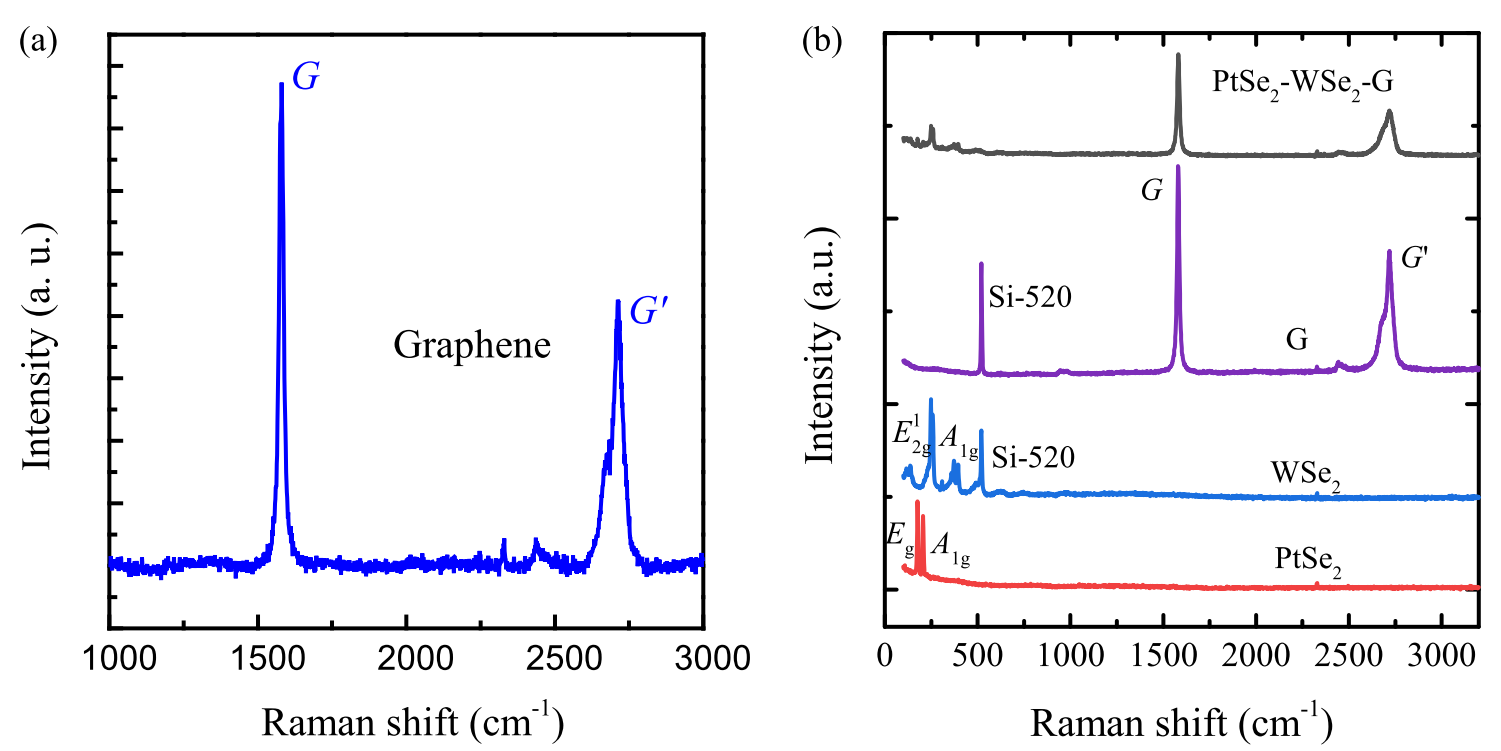


**Figure S1.** (a) Raman Spectrum of the ML-graphene in a typical G-WSe_2_-PtSe_2_ nBn heterostructure device. (b) Raman Spectra of a typical G-WSe_2_-PtSe_2_ nBn heterostructure device in the overlapped area and isolated graphene, WSe_2_, and PtSe_2_.


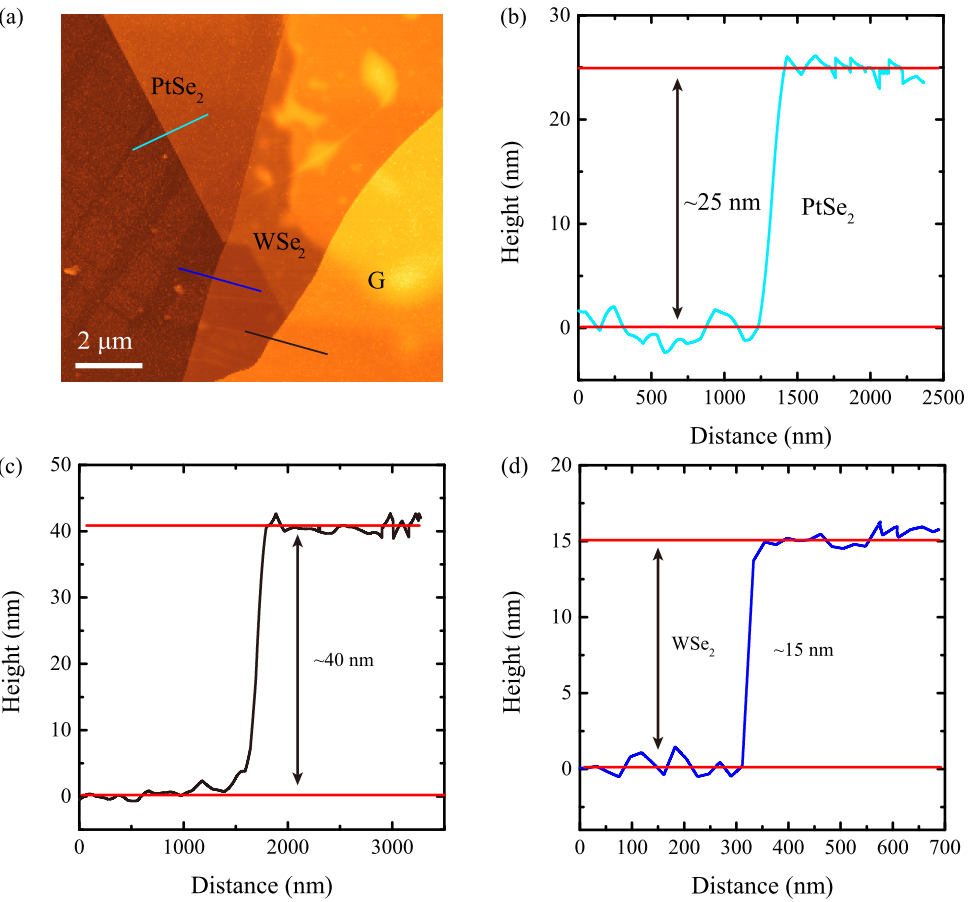


**Figure S2. The thicknesses of a typical G-WSe_2_-PtSe_2_ nBn device.** (a) AFM image of G-WSe_2_-PtSe_2_ heterodiode device. (b)-(d) The height profiles of the multilayer PtSe_2_, ML-graphene, and WSe_2_ flakes of a nBn device, respectively.


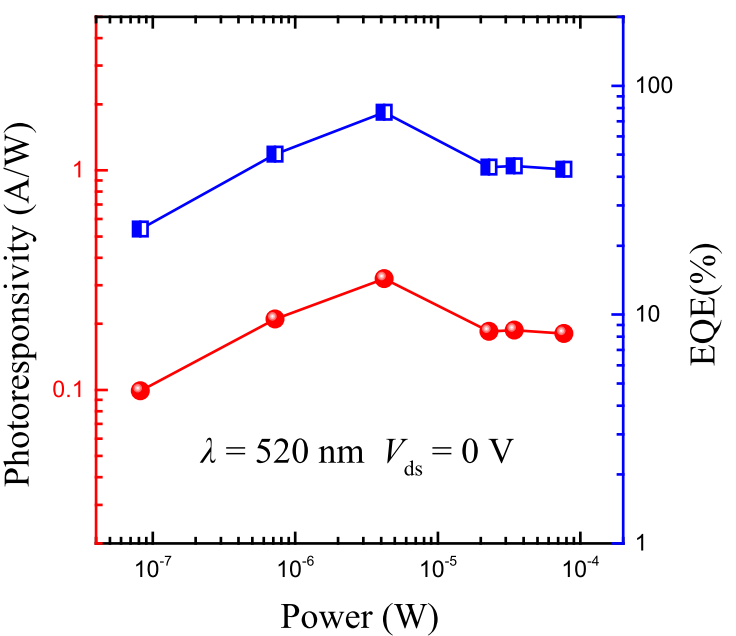


**Figure S3. Photoresponse of the G-WSe_2_-PtSe_2_ nBn device. Calculated *R* and EQE as a function of illumination power at 0 V bias.**


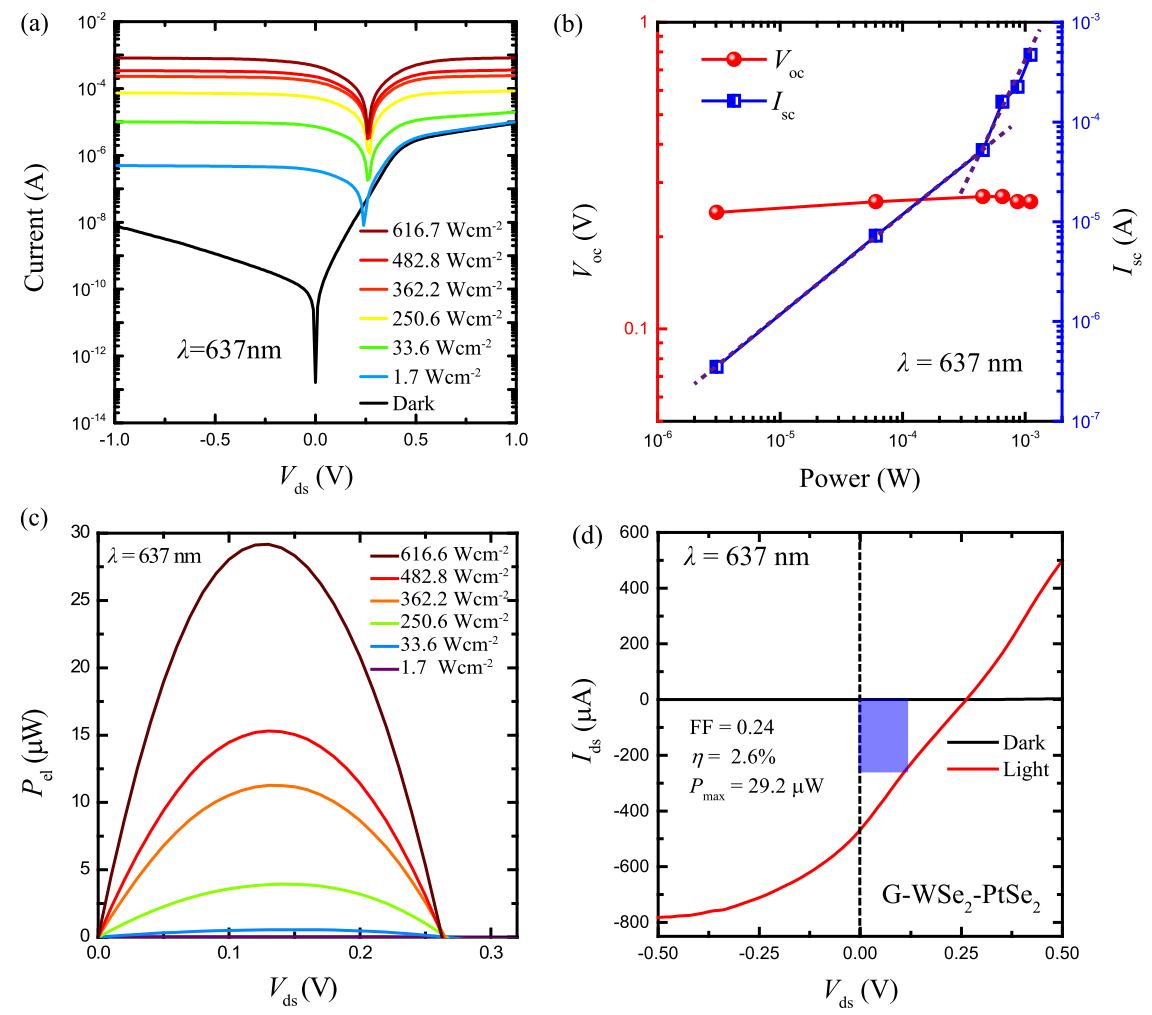


**Figure S4. The photoresponse of a typical G-WSe_2_-PtSe_2_ nBn device under 637 nm laser illumination.** (a) *I-V* curves of the G-WSe_2_-PtSe_2_ nBn device without and with various incident power of 637 nm laser. (b) Extracted *V*_OC_ and *I*_SC_ as a function of incident light power. (c) Electrical power *P*_el_ versus bias voltage with different illumination powers of 637 nm laser. (d) Photovoltaic response of the G-WSe_2_-PtSe_2_ nBn device with *FF* = 0.24 and energy conversion efficiency *η =* 2.6%, and maximum electrical power of 29.2 μW.


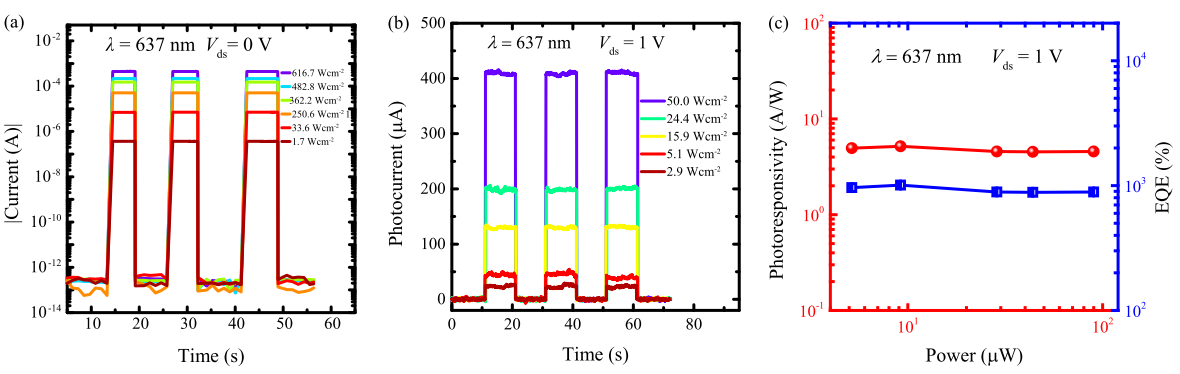


**Figure S5. The photoresponse of a typical G-WSe_2_-PtSe_2_ nBn device with a 637 nm laser.** (a) and (b) Time-resolved photoresponse of the G-WSe_2_-PtSe_2_ nBn device with different incident light powers of 637 nm laser at 0 V and 1 V biases, respectively. (b) Calculated *R* and EQE of the G-WSe_2_-PtSe_2_ nBn device as a function of incident light power at 1 V biases.


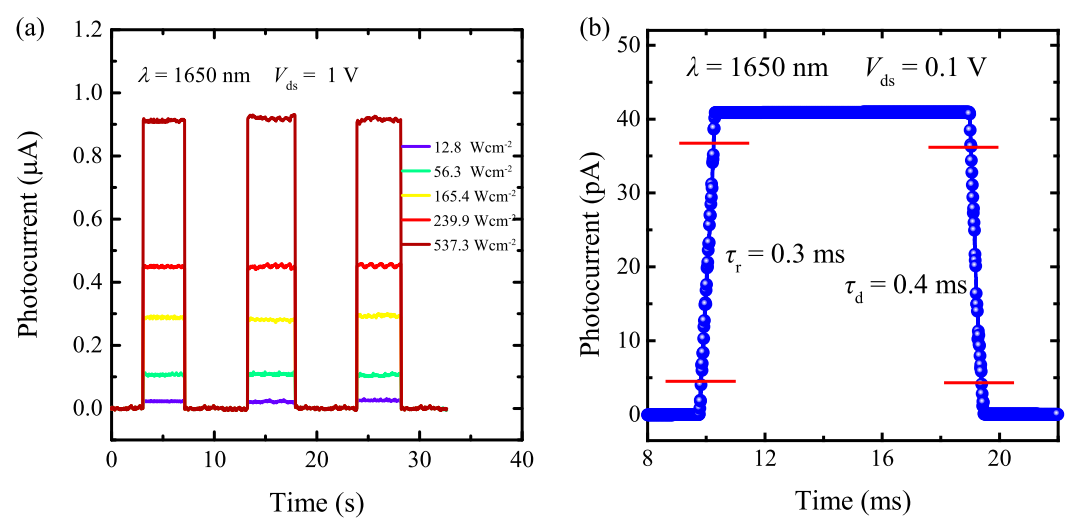


**Figure S6 The photoresponse of a typical G-WSe_2_-PtSe_2_ nBn device with 1650 nm nm laser.** (a) Time-resolved photoresponse of the G-WSe_2_-PtSe_2_ nBn device with a different incident light power of 1650 nm laser at 1 V biases. (b) photoresponse speed of the G-WSe_2_-PtSe_2_ nBn device with 1650 nm laser at 0.1 V bias.


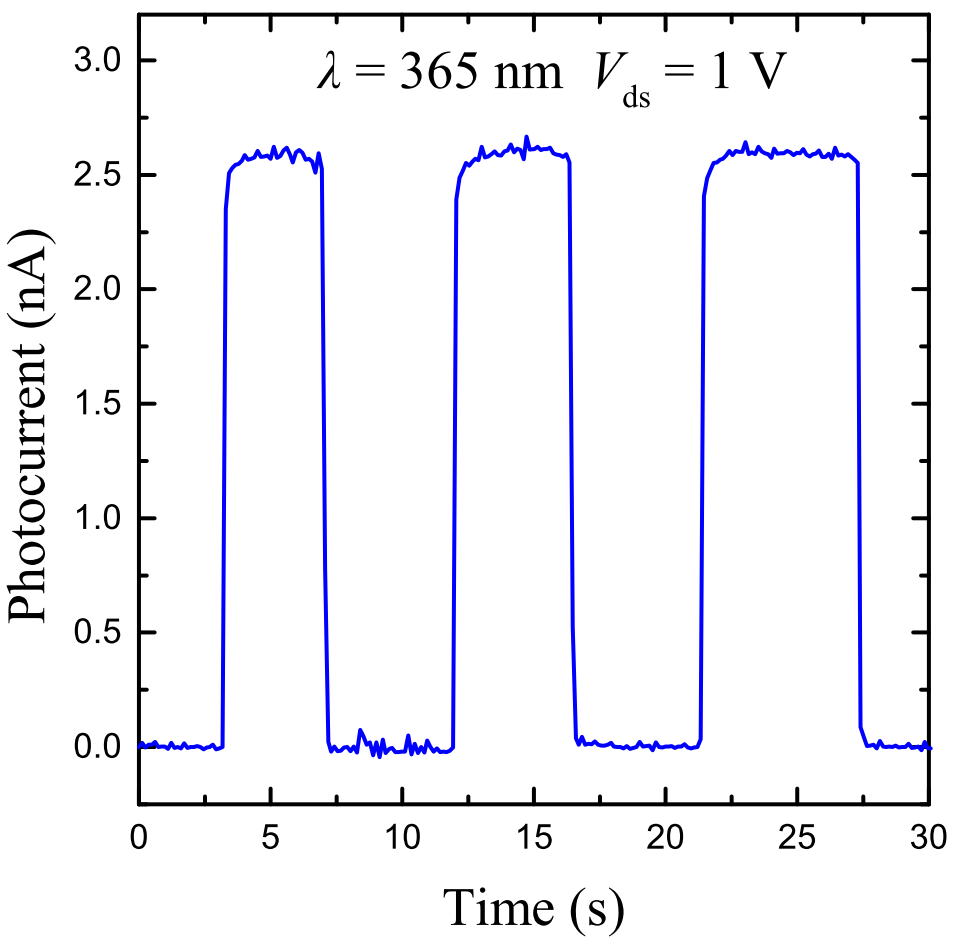


**Figure S7. The photoresponse of the G-WSe_2_-PtSe_2_ nBn device 365 nm illumination.** The temporal photoresponse of the G-WSe_2_-PtSe_2_ nBn device with 365 nm illumination at 1 V bias.


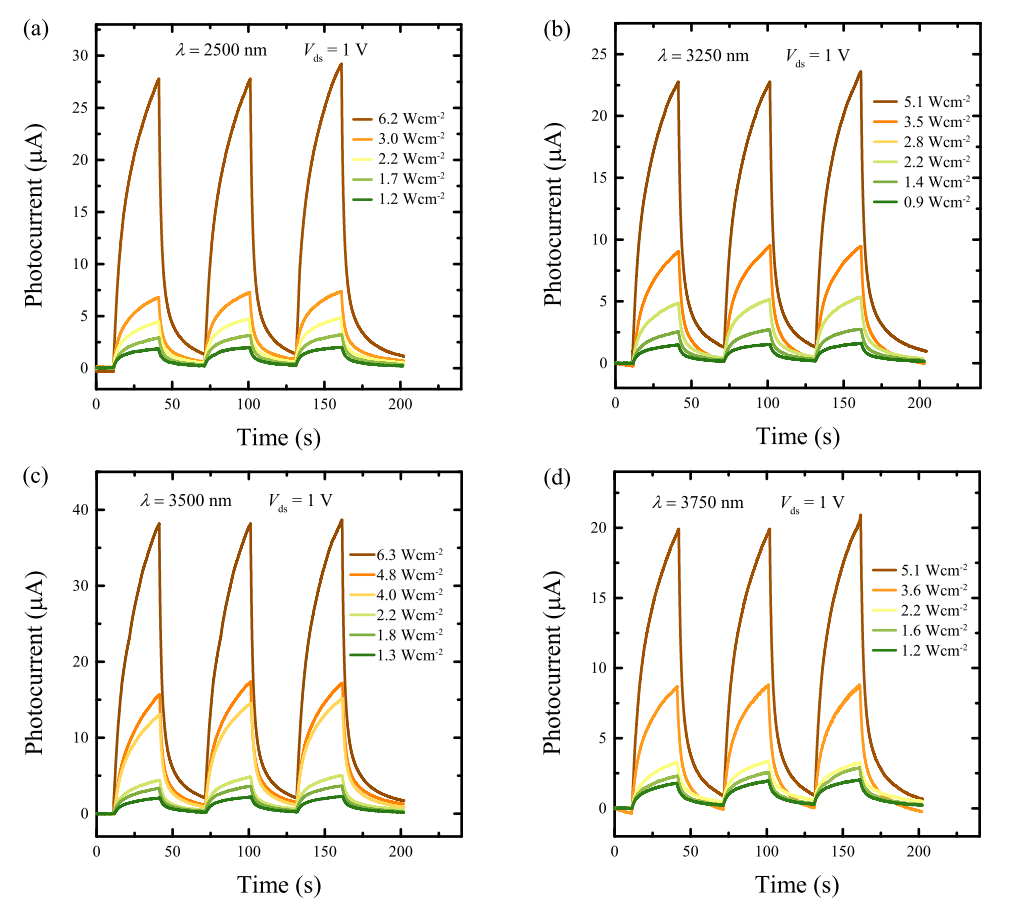


**Figure S8. Temporal photoresponse of the G-WSe_2_-PtSe_2_ nBn device in MWIR range.** (a)-(d) Temporal photoresponse of the G-WSe_2_-PtSe_2_ nBn device with different light powers of the 2500 nm, 3250 nm, 3500 nm, and 3750 nm lasers, respectively.


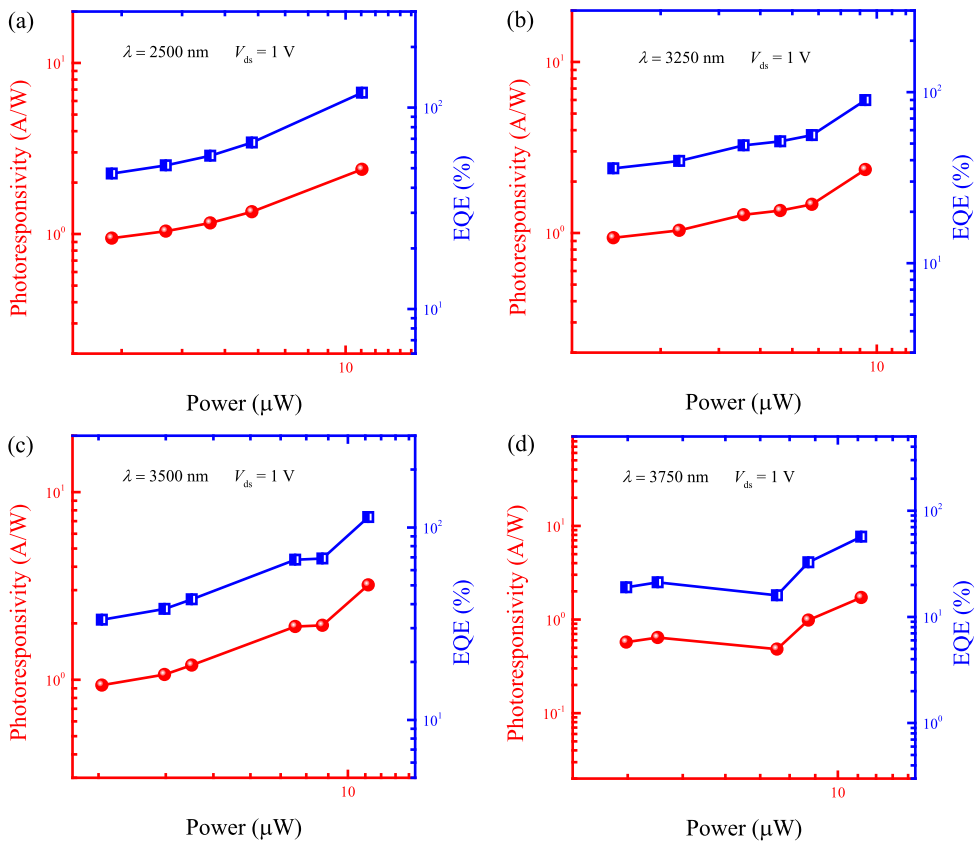


**Figure S9. The photoresponse of a typical G-WSe_2_-PtSe_2_ nBn device in the MWIR spectral range.** (a)-(d) Calculated *R* and EQE at 1 V bias versus incident light power of the 2500 nm, 3250 nm, 3500 nm, and 3750 nm lasers, respectively.


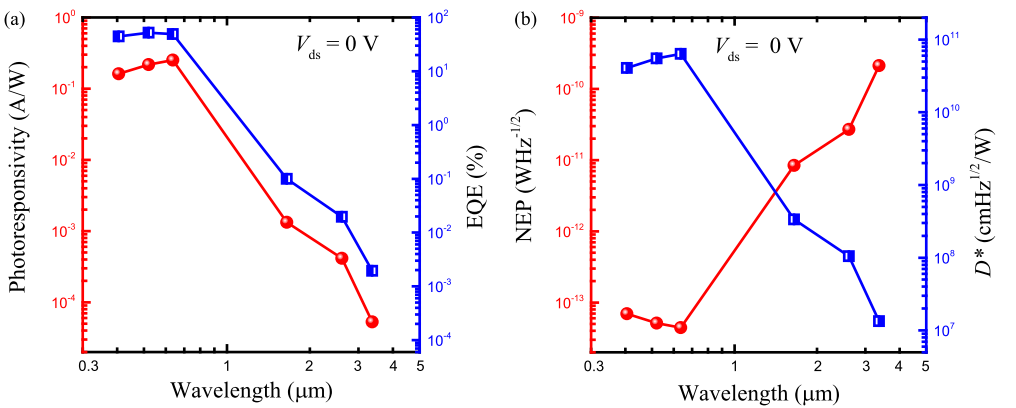


**Figure S10. Broadband photoresponse of the G-WSe_2_-PtSe_2_ nBn device. (a)** Calculated *R* and EQE as a function of wavelength at 0 V bias. (b) Extracted NEP and *D** of the G-WSe_2_-PtSe_2_ nBn device at 0 V bias.


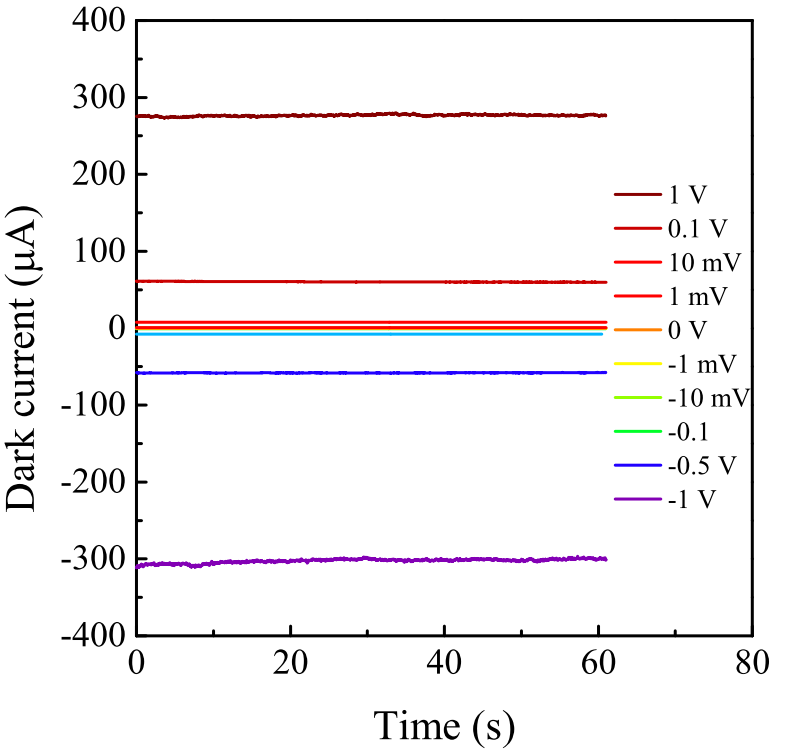


**Figure S11. The dynamic dark current of the G-WSe_2_-PtSe_2_ nBn device at different biases.**

Table S1. Summary of the performance of the 2D material photovoltaic response

| Structure | *λ*(nm) | *R* (A/W), EQE (0 V) | Time (s) | *V*_OC_ (V) | *η*, FF | *P*_el_-m | Ref. |
| --- | --- | --- | --- | --- | --- | --- | --- |
| Al-Doped BP | 1550 | 6.2 mA/W | 3, 10 ms | 140 mV | 0.66% | 54.3 pW | ^1^ |
| WSe_2_-2 *V*_g_ | 500-900 | 1.6×10^-2^ (-1 V) | - | 0.85 | 0.5%, 0.5 | 9 pW | ^2^ |
| BP-MoS_2_ | 633 | 0.418, 18% | - | 0.3 | 0.3%, 0.5 | 1.8 nW | ^3^ |
| BP-BN-2 *V*_g_ | 532-940 | 0.24×10^-3^, 0.102% | 1.3, 1.9 ms | 0.05 | 0.3 | 13 pW | ^4^ |
| WSe_2_-MoS_2_ | 590 | 11×10^-3^, 1.5% | - | 0.58 | 0.2%, 0.5 | 14 pW | ^5^ |
| WSe_2_-2 *V*_g_ | 442-940 | 0.7×10^-3^ |  | 0.5-0.7 | 0.01% | 0.17 nW | ^6^ |
| WSe_2_- 2 *V*_g_ | 522-755 | 0.21 (-2 V), 0.2% |  | 0.5-0.75 | 0.2% | 0.35 μW | ^7^ |
| MoS_2_-vertiacl | 655 | 0.03 | - | 0.6 | 0.4%, 0.22 | - | ^8^ |
| WSe_2_-MoS_2_ |  | - | - | 0.22 | 0.2%, 0.39 | - | ^9^ |
| WSe_2_-G | 532 | 6.62×10^-2^, 15% | 50 μs, 30 μs | 0.5 | 0.27%, | 0.46 μW | ^10^ |
| GaTe-MoS_2_ | 473 | 21.83 (1 V) | 195, 163 ms | 63 mV | 0.45%, 0.42 | 74 pW | ^11^ |
| Ag/MoS_2_/Pt | 532 | 0.0166 (0 V) | - | 1.02 | 0.61%, 0.47 | 3.7 nW | ^12^ |
| MoTe_2_/MoS_2_ | 637 | 0.046 | 60, 25 μs | 0.51 | - | - | ^13^ |
| MoTe_2_/MoS_2_ | 254-600 | 0.25, 56% | 2, 2 ms | 0.08 | 0.55%,0.35 | 6 pW | ^14^ |
| BP/GaAs | 514 | 0.037, 9.7% | - | 0.6 | 0.24%, 0.3 | 0.15 μW | ^15^ |
| MoSe_2_/GaN | 850 |  |  | 0.62 | 1.29%, 0.348 |  | ^16^ |
| MoS_2_/InP |  | - | - | 0.35 | 4.1%, 0.46 | - | ^17^ |
| ReS_2_/ReSe_2_ | 400-550 | 0.02, 4.76% | 0.4, 0.4 s | 0.175 | 0.48%, 0.37 | - | ^18^ |
| WSe_2_-Bi_2_Te_3_ | 1550 | 1.9×10^-3^ | 0.18, 0.21 ms | 0.25 | 4.39% | 1.5 nW | ^19^ |
| MoS_2_-CdTe | 200-1700 | 0.0366 | 43.7, 82.1 µs | - | - | - | ^20^ |
| MoS_2_-BP | 532-1550 | 0.153, 12% (3V) | 15, 70 µs | - | - | - | ^21^ |
| WSe_2_/GeSe | 520 | 6.2, 1490% (-1.5 V) | 30, 35.5 μs | - | - | - | ^22^ |
| MoS_2_/AsP | 520 | 0.3, 71% | 9, 5 µs | 0.61 | 9%, 0.5 | 0.39 μW | ^23^ |
| AsP/InSe | 520-1550 | 0.006, 1.5% | 217, 89 μs | 7.2 mV | - | 70 pW | ^24^ |
| PtSe_2_/Ge | 1550 | 0.16, 0.67% | 7.42, 16.7 μs | 0.602 | 0.67% | - | ^25^ |
| PtSe_2_/GaAs | 808 | 0.262 | 5.5, 6.5 μs | - | - | - | ^26^ |
| WSe_2_-WSe_2_ | 532 | 0.011, 2.6% | 0.18, 0.22 ms | 0.49 | 0.13%, 0.31 | - | ^27^ |
| **G-WSe_2_-PtSe_2_** | **365 nm-10.6 μm** | **0.75, 146.1%** | **699, 452 ns** | **0.28** | **4.87%, 0.41** | **29.2 μW** | **This work** |

**References**

1. Liu, Y., Cai, Y., Zhang, G., Zhang, Y. & Ang, K. Al-Doped Black Phosphorus p-n Homojunction Diode for High Performance Photovoltaic. *Adv. Funct. Mater.* **27**, 1604638 (2017).

2. Pospischil, A., Furchi, M. M. & Mueller, T. Solar-energy conversion and light emission in an atomic monolayer p-n diode. *Nat. Nanotechnol.* **9**, 257-261 (2014).

3. Deng, Y. et al. Black Phosphorus-Monolayer MoS_2_ van der Waals Heterojunction p-n Diode. *ACS Nano*. **8**, 8292-8299 (2014).

4. Buscema, M., Groenendijk, D. J., Steele, G. A., van der Zant, H. S. J. & Castellanos-Gomez, A. Photovoltaic effect in few-layer black phosphorus PN junctions defined by local electrostatic gating. *Nat. Commun.* **5**, 4651 (2014).

5. Furchi, M. M., Pospischil, A., Libisch, F., Burgdoerfer, J. & Mueller, T. Photovoltaic Effect in an Electrically Tunable van der Waals Heterojunction. *Nano Lett.* **14**, 4785-4791 (2014).

6. Groenendijk, D. J. et al. Photovoltaic and Photothermoelectric Effect in a Double-Gated WSe_2_ Device. *Nano Lett.* **14**, 5846-5852 (2014).

7. Baugher, B. W. H., Churchill, H. O. H., Yang, Y. & Jarillo-Herrero, P. Optoelectronic devices based on electrically tunable p-n diodes in a monolayer dichalcogenide. *Nat. Nanotechnol.* **9**, 262-267 (2014).

8. Li, H. et al. Ultimate thin vertical p-n junction composed of two-dimensional layered molybdenum disulfide. *Nat. Commun.* **6**, 6564 (2015).

9. Li, M. Y. et al. Epitaxial growth of a monolayer WSe_2_-MoS_2_ lateral p-n junction with an atomically sharp interface. *Science*. **349**, 524-528 (2015).

10. Gao, A. et al. Gate-tunable rectification inversion and photovoltaic detection in graphene/WSe_2_ heterostructures. *Appl. Phys. Lett.* **108**, 223501 (2016).

11. Wang, F. et al. Tunable GaTe-MoS_2_ van der Waals p-n Junctions with Novel Optoelectronic Performance. *Nano Lett.* **15**, 7558-7566 (2015).

12. Liu, Y. et al. Approaching the Schottky–Mott limit in van der Waals metal-semiconductor junctions. *Nature*. **557**, 696-700 (2018).

13. Chen, Y. et al. High-Performance Photovoltaic Detector Based on MoTe_2_/MoS_2_ Van der Waals Heterostructure. *Small*. **14**, 1703293 (2018).

14. Wu, E. et al. Photoinduced Doping To Enable Tunable and High-Performance Anti-Ambipolar MoTe_2_ /MoS_2_ Heterotransistors. *ACS Nano*. **13**, 5430-5438 (2019).

15. Gehring, P., Urcuyo, R., Duong, D. L., Burghard, M. & Kern, K. Thin-layer black phosphorus/GaAs heterojunction p-n diodes. *Appl. Phys. Lett.* **106**, 233110 (2015).

16. Chen, Z. et al. Wafer-Size and Single-Crystal MoSe_2_ Atomically Thin Films Grown on GaN Substrate for Light Emission and Harvesting. *ACS Appl. Mater. Interfaces*. **8**, 20267-20273 (2016).

17. Wang, P. et al. Enhanced monolayer MoS_2_/InP heterostructure solar cells by graphene quantum dots. *Appl. Phys. Lett.* **108**, 163901 (2016).

18. Cho, A., Namgung, S. D., Kim, H. & Kwon, J. Electric and photovoltaic characteristics of a multi-layer ReS_2_/ReSe_2_ heterostructure. *APL Mater.* **5**, 76101 (2017).

19. Liu, H. et al. Self-Powered Broad-band Photodetectors Based on Vertically Stacked WSe_2_/Bi_2_Te_3_ p-n Heterojunctions. *ACS Nano*. **13**, 13573-13580 (2019).

20. Wang, Y. et al. A room-temperature near-infrared photodetector based on a MoS_2_/CdTe p-n heterojunction with a broadband response up to 1700 nm. *J. Mater. Chem. C*. **6**, 4861-4865 (2018).

21. Ye, L., Li, H., Chen, Z. & Xu, J. Near-Infrared Photodetector Based on MoS_2_/Black Phosphorus Heterojunction. *ACS Photonics*. **3**, 692-699 (2016).

22. Wei, X., Yan, F., Lv, Q., Shen, C. & Wang, K. Fast gate-tunable photodetection in the graphene sandwiched WSe_2_/GaSe heterojunctions. *Nanoscale*. **9**, 8388-8392 (2017).

23. Wu, F. et al. High efficiency and fast van der Waals hetero-photodiodes with a unilateral depletion region. *Nat. Commun.* **10**, 4663 (2019).

24. Wu, F. et al. AsP/InSe Van der Waals Tunneling Heterojunctions with Ultrahigh Reverse Rectification Ratio and High Photosensitivity. *Adv. Funct. Mater.* **29**, 1900314 (2019).

25. Lu, Y. et al. Construction of PtSe_2_/Ge heterostructure-based short-wavelength infrared photodetector array for image sensing and optical communication applications. *Nanoscale*. **13**, 7606-7612 (2021).

26. Zeng, L. et al. Fast, Self-Driven, Air-Stable, and Broadband Photodetector Based on Vertically Aligned PtSe_2_/GaAs Heterojunction. *Adv. Funct. Mater.* **28**, 1705970 (2018).

27. Tan, C. et al. A Self-Powered Photovoltaic Photodetector Based on a Lateral WSe_2_-WSe_2_ Homojunction. *ACS Appl. Mater. Interfaces*. **12**, 44934-44942 (2020).
